# Radiomics Analysis of Deltoid Muscle on Pre-Operative Shoulder Imaging for Reverse Total Shoulder Arthroplasty

**DOI:** 10.51894/001c.143951

**Published:** 2025-09-30

**Authors:** Henry J. Lee

**Affiliations:** 1 College of Osteopathic medicine Michigan State University

## 29

### INTRODUCTION

Reverse total shoulder arthroplasty (rTSA) is a common procedure used to correct rotator cuff pathologies. Postoperative risks include pain and neurological complications. Some studies have demonstrated that deltoid muscle volume has an association with rTSA outcomes.

### OBJECTIVES

In this study, radiomics and artificial intelligence (AI) techniques are used to characterize preoperative deltoid muscle volume from routine CT imaging and low-cost US assessment with the future goal of determining if this information can predict postoperative shoulder function.

### METHDOS

This IRB-approved retrospective study includes 75 patients aged 18-89 that underwent US and CT of the shoulder within 2 years prior to a rTSA between 1/1/2014 to 12/31/2022. US images of the anterior deltoid are uniformly captured by centering the US probe along the long head of the biceps tendon. The deltoid muscle in both modalities were manually segmented by trained medical students or residents and analyzed with AI techniques for summary shape and intensity features to determine if US can provide similar muscle information as CT.

### RESULTS

The current cohort includes 75 subjects with CT and/or US exams. CT revealed a mean deltoid volume of 293+/-106 cm3 and 1.6% average decrease in volume for each year of age. The muscle was also characterized by apparent mass from a calculation of approximate density (estimated from mean CT number) times volume, yielding a mean of 303+/-111g. From 33 subjects with segmented deltoid US, the mean deltoid thickness was calculated to be 0.91+/-0.23 cm. The linear relationship between CT derived volume and US derived deltoid thickness was determined with a correlation coefficient of 0.45 and a standard error of the estimate of 89 cm3, suggesting that US can be used as a weak predictor of deltoid volume.

### CONCLUSION

This preliminary analysis of preoperative imaging for rTSA suggests that CT can be used to characterize deltoid shape and mass. Likewise, US provides correlative data that may be used to predict deltoid volume and mass. Future work is needed to evaluate US ability to predict CT information and postoperative outcomes, and its potential to provide additional insight into the postoperative management of patients.

